# Physical evidence of meminductance in a passive, two-terminal circuit element

**DOI:** 10.1038/s41598-022-24914-y

**Published:** 2023-02-01

**Authors:** Abhiram Dinavahi, Alexandre Yamamoto, H. Rusty Harris

**Affiliations:** Department of Electrical and Computer Engineering, Texas A&M University, College Station, TX 77840 USA

**Keywords:** Electrical and electronic engineering, Applied physics

## Abstract

The first intentional memristor was physically realized in 2008 and the memcapacitor in 2019, but the realization of a meminductor has not yet been conclusively reported. In this paper, the first physical evidence of meminductance is shown in a two-terminal passive system comprised primarily of an electromagnet interacting with a pair of permanent magnets. The role of series resistance as a parasitic component which obscures the identification of potential meminductive behavior in physical systems is discussed in detail. Understanding and removing parasitic resistance as a “resistive flux” is explored thoroughly, providing a methodology for extracting meminductance from such a system. The rationale behind the origin of meminductance is explained from a generalized perspective, providing the groundwork that indicates this particular element is a realization of a fundamental circuit element. The element realized herein is shown to bear the three required and necessary fingerprints of a meminductor, and its place on the periodic table of circuit elements is discussed by extending the genealogy of memristors to meminductors.

## Introduction

In his seminal 1971 paper^1^, Leon Chua observed that while the resistor, the capacitor and the inductor were respectively defined by current–voltage, charge–voltage, and current-flux relationships, a circuit element defined by charge-flux relationship was missing. This led him to conceive of the fourth fundamental circuit element, the memristor which was characterized by a constitutive relationship between charge and flux. In 1977, Chua defined the larger class of memristive systems^2^ and updated the defining feature of a memristor to be a “pinched hysteresis” curve in the current–voltage plane. He later went on to develop the genealogy of memristors^3^, with the original idea of charge-flux relationship only defined to be a requirement for ideal memristors and not for generic and extended memristors. The idea of a constitutive relationship in the (v^(α)^ − i^(β)^) plane being the distinguishing feature of an ideal circuit element- where v^(α)^(t) is defined by (1) and α, β are integers- further led to the theoretical possibility of infinitely many such elements, populating a doubly periodic table of fundamental circuit elements^4,5^.

1 $$  {v^{{\left( \alpha  \right)}} \left( t \right) \triangleq \left\{ {\begin{array}{*{20}l}    {\frac{{d^{\alpha } v\left( t \right)}}{{dt^{\alpha } }},} \hfill & \quad {if\,\alpha  > 0} \hfill  \\    {v\left( t \right),} \hfill & \quad {if\,\alpha  = 0} \hfill  \\    {\smallint _{{ - \infty }}^{t} \smallint _{{ - \infty }}^{{\tau _{2} }}  \ldots \smallint _{{ - \infty }}^{{\tau _{{ - \alpha }} }} v\left( {\tau _{{ - \alpha }} } \right)d\tau _{{ - \alpha }} d\tau _{{ - \alpha  - 1}}  \ldots d\tau _{1} ,} \hfill & \quad {if\,\alpha  < 0} \hfill  \\   \end{array} } \right.}  \\  $$

Leon Chua also notes in his 1971 paper that “while a memristor behaves as an ordinary resistor at any given instant of time, t_0_, its resistance (conductance) depends on the complete past history of the memristor current (voltage)”. This being a mathematical description, can be generalized and used as the guiding principle for the physical realization of any fundamental circuit element. Of particular interest among such elements are a capacitor whose capacitance (elastance) depends on the history of its voltage (charge), called the memcapacitor, and an inductor whose inductance (reluctance) depends on the history of its current (flux), called the meminductor^6^. While the memristor has been physically realized in 2008^7^ and the memcapacitor in 2019^8^, the meminductor has remained elusive so far.

It is important to recognize the contemporary debate on the utility of applying Chua’s mathematical model to modern 2-terminal elements. Indeed, for memristive elements driven by oxygen vacancy transport, the impact of accurate ionic diffusion models on the state variable are still debated, not to mention the stored energy thermodynamic arguments used against the classification of resistive memory as a memristor. However, completing the mem-element mosaic by accurately mapping 2-terminal elements into the model is critical to providing tools to device engineers and scientists in important research areas such as neuromorphic computing and memory architecture. Therefore, the discovery and understanding of a meminductive element is vital to the scientific discussion of device classification and the furtherance of important emerging technology areas.

Much like memcapacitive^9^ devices, meminductive devices, owing to their inherent energy storage properties, could potentially offer lower static power consumption than memristive devices for large-scale, energy-efficient neuromorphic computing applications. Furthermore, dynamical circuit applications of mem-elements involving local activity, edge of chaos, and resulting persistent dynamics^10–13^ adds more value to a physical implementation of a meminductor. However, despite work being published on SPICE modeling of meminductors^14^ and potential ways of achieving meminductance in physical systems^15^, its realization is yet to be reported. A previous publication^16^ claiming to have realized a meminductor fails to grasp its essence as a two-terminal circuit element and reports pinched hysteresis in the flux-current behavior not between the two terminals of the element but elsewhere. In this paper, we report the first true physical evidence of meminductance in a passive two-terminal system.

## Generalized mathematical description of circuit elements

**Figure 1:**
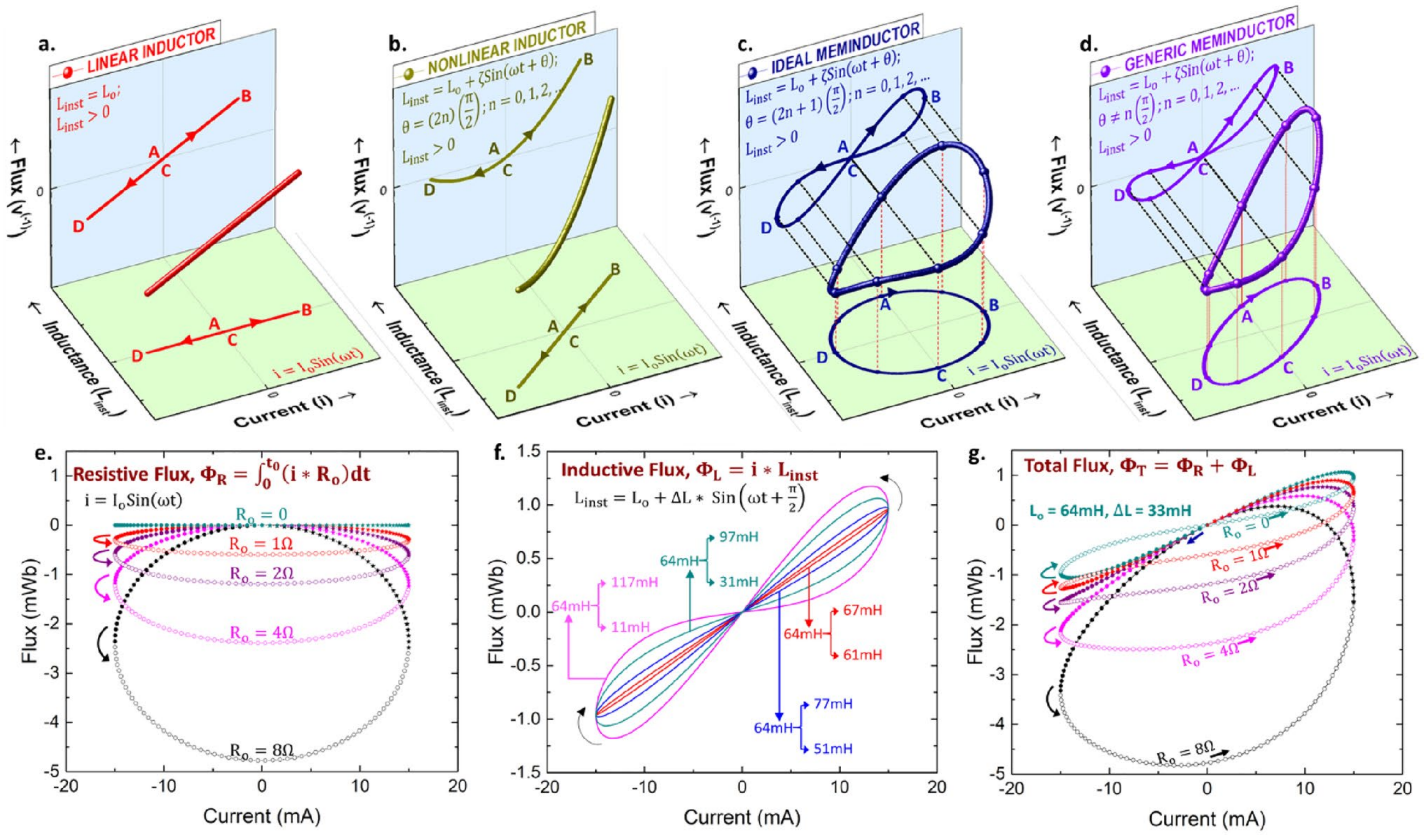
Physical realization of a meminductor: approach and challenges. (**a**–**d**) Choice of the ordered triple as (i, v^(−1)^, L_inst_) identifies the two-terminal circuit element as a current sourced inductor. For a sinusoidally varying i(t), a constant L_inst_ yields a linear inductor (**a**), whereas a time variation induced in L_inst_ due to its state-dependence introduces nonlinearity. A sinusoidal fit for L_inst_ is chosen here for clear illustration. The phase difference between i(t) and L_inst_ being even multiples of $\frac{\pi }{2}$ results in an ideal non-linear inductor (**b**), a phase difference of odd multiples of $\frac{\pi }{2}$ results in an ideal meminductor (**c**), and any other phase difference results in a generic meminductor (**d**). (**e–g**) Impact of series resistance on pinched hysteresis behavior of a meminductor: illustrated for a winding driven by a sinusoidal current signal, i(t) with I_o_ = − 15 mA and f = 8 Hz. Resistive flux, Φ_R_: series resistance, R_o_ results in a right-handed ellipse in the (i, Φ_R_) plane (**e**), Inductive flux, Φ_L_: winding inductance, L_inst_ results in a pinched hysteresis curve in the (i, Φ_L_) plane, illustrated for L_o_ = 64 mH with ΔL = 53 mH (magenta), 33 mH (cyan), 13 mH (blue), and 3 mH (red) (**f**), Total flux, Φ_T_, calculated as the sum of resistive and inductive flux components illustrates the pinch point of the meminductive response of a winding with (L_o_, ΔL) = (64 mH, 33 mH) made to disappear by series resistances 2 Ω and greater at 8 Hz (**g**).

The conventional Ohm’s law, $\mathrm{v}=\mathrm{i}*\mathrm{R}$, can be represented as an ordered triple (i, v, R) and generalized to describe all three traditional circuit elements through appropriate choice of the constituents of the equation: the resistor described by (i, v, R) and/or (v, i, G), the capacitor by (v, i^(−1)^, C) and/or (i^(−1)^, v, C^−1^), and the inductor by (i, v^(−1)^, L) and/or (v^(−1)^, i, L^−1^). Table 1 summarizes the α/β notation of current and voltage in the three traditional circuit elements. This table also maps the α/β notation to classically understood variables for each of the elements. The following discussion focuses on a current-sourced inductor for a periodic sourcing function, i(t), with zero mean and zero initial condition, i(0) = 0, and can be easily extended to any of the six ordered triple combinations shown in Table 1. A constant, state-independent instantaneous relationship between i(t) and v^(−1)^(t) describes the classical inductor, with the slope of the inductor’s characteristic i − v^(−1)^ curve being the element’s familiar characteristic inductance. The linear nature of the classical inductor can be seen in the flux-current curve of Fig. 1a.

**Table 1:**
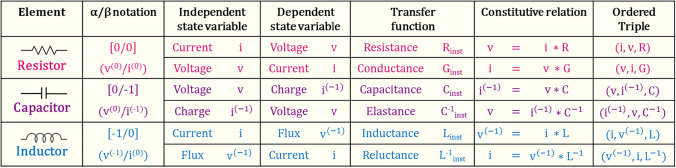
Generalized Ohm’s law for the traditional fundamental circuit elements.

Choice of the independent and dependent state variables and the associated transfer function in the generalized version of Ohm’s law to result in the three traditional fundamental circuit elements: a resistor, a capacitor, and an inductor.

The first step in describing the “mem”-version of any of these elements is to underscore that the transfer function, what is classically thought of as L for an inductor, is not necessarily constant, and how it functionally varies can be complex. The simplest case of the transfer function varying based on the condition of a state variable is a non-linear inductor, where the nonlinearity results from a single-valued dependence of the instantaneous inductance on the sourced current. This single-valued dependence is illustrated in Fig. 1b with a linear relationship (for convenience) between inductance and current, and this temporal variation results in a non-linear relationship between current and flux. The generalized Ohm’s law description, with the (i, v^(−1)^, L_inst_) triple, still applies and it follows that the flux-current relationship is single-valued, with the flux becoming zero whenever the current is zero. Hence, an ideal current-sourced nonlinear inductor is defined by

2 $$\begin{array}{c}{v}^{\left(-1\right)} = {L}_{inst}\left({i}^{\left(0\right)}\right)*{i}^{\left(0\right)} .\end{array}$$

(Details of the oscillatory procession to/from points B and D of the transfer function and its phase relationship in a Lissajous framework are given in Supplemental Material.)

The phenomenological cause of a transfer function’s variation constitutes a state variable, and this variable being different from the sourcing function results in a multi-valued relationship between the sourcing function and the transfer function, and thereby, mem-properties. It is usually extremely difficult to represent such state variable(s) fully mathematically, and this is the reason the discovery of memristors (and recently, memcapacitors) was so elusive until modern times. Meminductive properties are observed when a state-dependence of inductance makes it multivalued in current. However, the meminductor can still be described using the same general equation as the linear version, complete with an ordered triple, and is distinguished from its base-counterpart by the transfer function having a multi-valued dependence on the independent state variable.

A temporal variation in L_inst_ (with the same frequency as i(t) or a higher harmonic) which is not single-valued in i(t) results in a multi-valued hysteretic curve in the i − v^(−1)^ plane. The hallmark observation of a pinched hysteresis curve passing through the origin in the i − v^(−1)^ plane helps define the generic meminductor, as seen in Fig. 1d. A generic meminductor can be described by

3 $$\begin{array}{c}{v}^{\left(-1\right)}={L}_{inst}\left(s\left(t\right)\right)*{i}^{\left(0\right)},\end{array}$$

4 $$\begin{array}{c}\text{where}\, \frac{ds}{dt}=f\left(s,{i}^{\left(0\right)}\right) .\end{array}$$

here, s(t) is the state variable, and its time dependence can be mapped to the functional properties of the current and the phenomenon that causes the state variable through (4). A generic meminductor can have i^(−1)^ be zero for a non-zero value of v^(−2)^ and v^(−2)^ be zero for a non-zero value of i^(−1)^. Further, both i^(−1)^ and v^(−2)^ can be multi-valued functions of each other^17^.

A special case of such variation is a single valued dependence between L_inst_ and i^(−1)^ corresponding to an ideal meminductor. This dependence can be interpreted to be the combined result of individual single valued dependencies between L_inst_ and a state variable s(t), and between s(t) and i^(−1)^(t). Hence, an ideal meminductor can be synthesized by introducing a single-valued dependence between the instantaneous inductance, L_inst_ and charge, i^(−1)^(t) such that the general equation reduces to (5). An ideal current sourced meminductor, besides exhibiting a pinched hysteresis curve passing through the origin in the i − v^(−1)^ plane, is also characterized by v^(−2)^ being single valued in i^(−1)^. Further, zero initial conditions force v^(−2)^ to be zero whenever i^(−1)^ becomes zero. These relationships for an ideal meminductor can be obtained by integrating both sides of (5) over time and then be rearranged as shown in (6)

5 $$\begin{array}{c}{v}^{\left(-1\right)}={L}_{inst}\left({i}^{\left(-1\right)}\right)*{i}^{\left(0\right)}\end{array}$$

6 $$\begin{array}{c}{v}^{\left(-2\right)}={L}_{{\left(-1\right)}_{inst}}\left({i}^{\left(-1\right)}\right)*{i}^{\left(-1\right)}\end{array}$$

The corresponding equations for the (v^(−1)^, i, L^−1^_inst_) ordered triple are given in the supplementary information.

## Physical realization of a meminductor and impact of parasitic series resistance

The phase difference between i(t) and L_inst_ being even multiples of π/2 (i.e., 0, π, 2π, …) describes a single valued dependence between i(t) and v^(−1)^(t) and thereby, an inductor, as shown in Fig. 1a,b. On the other hand, a phase difference of odd multiples of π/2 (i.e., π/2, 3π/2, …) describes a single valued dependence between i^(−1)^(t) (i.e., charge) and L_inst_, and thus, an ideal meminductor. Any phase difference between i(t) and L_inst_ besides integral multiples of π/2 (i.e., 0, π/2, π, 3π/2, 2π, …) describes a generic meminductor. Ideal and generic meminductors hence have multiple values of v^(−1)^(t) for a given value of i(t) thus resulting in hysteresis lobes pinched at the origin and are shown in Fig. 1c,d, respectively. Therefore, the physical realization of an ideal current-sourced meminductor requires an inductor whose instantaneous inductance monotonically increases or decreases as long as the polarity of the sourced current signal does not change, thus resulting in a 90° phase difference between i and L_inst_. Achieving such dependence in an inductor with a two-terminal passive configuration outlines the goal of this work.

The AC response of a two-terminal winding consists not only of an inductive component, but also of parasitic resistive and capacitive components. The series parasitic resistance- primarily from the coil winding- is particularly notorious for swamping the inductive response at low frequencies. This complicates physical realization of a meminductor since the electrical response of a mem-element converges towards that of its respective element as the frequency increases^18^. Hence, high frequencies are required for the inductive component of impedance to be more dominant than the resistive component, but the inductive component fails to manifest as meminductance at such frequencies. This requirement of low frequency operation thereby necessitates means to either eliminate the series resistance or extract the meminductive component obscured by the more dominant resistive component. This work employs the latter strategy.

It is useful to note here that parallel resistance plays a similar role in the physical realization of memcapacitors as series resistance does in meminductors: for a memcapacitor, high capacitive impedance at low frequencies results in the resistive branch drawing most current and thereby being the dominant component swamping potential memcapacitance. On the other hand, high frequency operation destroys memcapacitance and the device behaves as a linear capacitor.

A current sourced winding with a time varying instantaneous inductance, L_inst_(t) and effective series resistance, R_o_ has been considered to study the mechanism of series resistance swamping evidence of meminductive behavior. A phase difference of 90° is enforced between i(t) and L_inst_(t) as described by the equations in Fig. 1e,f. Defining flux as the voltage integrated over time allows the total flux, Φ_T_ to be expressed as the sum of the resistive and inductive flux components, Φ_R_ and Φ_L_, respectively as shown in Eqs. (7)–(9).

7 $$\begin{array}{c}{\Phi }_{R}\left(t\right){\left.\right|}_{t={t}_{0}}= {\int }_{-\infty }^{{t}_{0}}i\left(t\right)*{R}_{o}dt={\int }_{0}^{{t}_{0}}i\left(t\right)*{R}_{o}dt\end{array}$$

8 $$\begin{array}{c}{\Phi }_{L}\left(t\right){\left.\right|}_{t={t}_{0}}=i\left({t}_{0}\right)*{L}_{inst}\left({t}_{0}\right) \end{array}$$

9 $$\begin{array}{c}{\Phi }_{T}\left(t\right){\left.\right|}_{t={t}_{0}}=\left({\int }_{0}^{{t}_{0}}i\left(t\right)*{R}_{o}dt\right)+\left(i\left({t}_{0}\right)*{L}_{inst}\left({t}_{0}\right)\right)\end{array}$$

A current signal with I_o_ = − 15 mA and f = 8 Hz results in the plots shown in Fig. 1e–g, with Φ_R_ being a unipolar ellipse with a counter-clockwise direction everywhere (Fig. 1e) and Φ_L_ being a bipolar pinched hysteresis curve with counter-clockwise and clockwise directions in the first and third quadrants, respectively (Fig. 1f). Such contrasting directions of Φ_R_ and Φ_L_ result in the pinch point in the total flux curve drifting away from the origin as R_o_ is increased and eventually disappearing as shown in Fig. 1g. The pinch point of a 64 mH inductor with ΔL of 33 mH at 8 Hz can be made to completely disappear by a series resistance as small as 2 Ω thus underlining a serious complication in the search for meminductive behavior in practical physical devices. For higher R_o_, the total flux takes the shape of a distorted ellipse.

Knowledge of the current sourced and a priori measurement of series winding resistance allows the calculation of the resistive voltage across the winding, and thereby resistive flux. On the other hand, since the total voltage can be measured, the total flux can also be calculated. The hidden meminductive behavior of systems where the series resistance cannot be eliminated can hence be extracted by subtracting the resistive flux from the total flux to obtain the meminductive flux at every instant. This technique is adopted in this study.

To test the validity of this idea, an axi-symmetric COMSOL Multiphysics^®^ model of a winding with a movable ferromagnetic core^19^ has been developed (Supplementary Figure-3) such that relative motion between the winding and the core can be mathematically defined to enable controlled time variation of L_inst_. As the core slides into and out of the volume of winding, L_inst_ gradually increases and decreases, respectively. Therefore, by forcing a 90° phase difference between the core displacement, d(t) and the current sourced, i(t), the same phase difference can be extended to L_inst_ and i(t). This setup results in meminductive behavior since the quasi-static response of the system is inductive, with the value of the inductance at any instant depending on the history of the current sourced. However, the system only serves as a meminductor emulator, since the core displacement is not enforced by the sourced current but is independently controlled, thereby making it a 3-terminal, potentially active device, in direct contradiction with the requirement that any fundamental circuit element be passive and consist only of two terminals.

Simulation parameters have been described in Supplementary Section-3 and results from Supplementary Figure-3(b) indicate the total flux being almost indistinguishable from an ellipse, thus indicating an overwhelming dominance of resistive behavior over inductive behavior at a frequency of 8 Hz. However, subtracting resistive flux from the total flux reveals pinched hysteresis response as shown in Supplementary Figure-3(c), confirming hidden meminductive behavior despite the (mem)inductive flux being over two orders of magnitude smaller than the total flux.

## Experimental setup, results, and discussion

**Figure 2:**
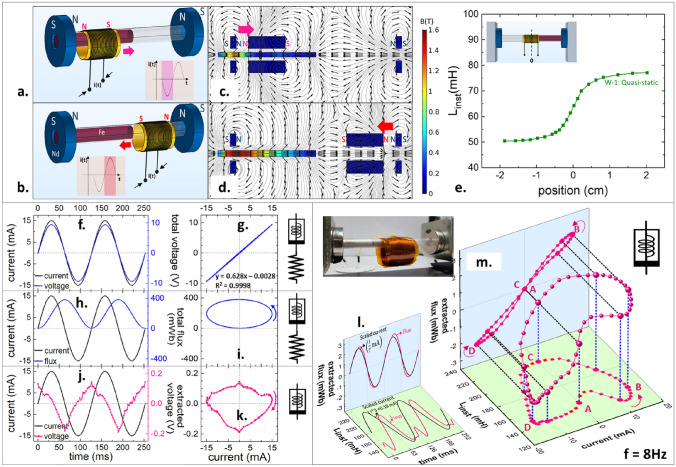
Experimental setup and results: (**a**–**d**) Two neodymium permanent magnets with like poles facing each other connected by a smooth shaft along which the fabricated winding can move freely; the core volume is partially filled by a ferromagnetic rod. Alternating negative (**a**) and positive (**b**) half-cycles in the sourced current result in alternating magnetic poles on the winding thus periodically switching the direction of force exerted on it by the permanent magnets. A periodic back and forth winding motion results from this force profile. Simulated magnetic field pattern for winding positioned as in (‘**a**’) and (‘**b**’) shown in (**c**,**d**), respectively. (**e**) Quasi-static inductance measurements on W-1 as a function of the position of the winding relative to the core; zero-reference position in the setup shown in the inset. (**f**,**g**) Total voltage measured across the terminals of the winding, W-1 by sourcing sinusoidal current, (**h**,**i**) Total flux calculated as the time integral of the total voltage measured, (**j**,**k**) (Mem)inductive voltage extracted from the total voltage by subtracting resistive voltage, (**l**,**m**) Extracted (mem)inductive flux calculated as the time integral of the extracted (mem)inductive voltage, and instantaneous dynamic inductance calculated as the ratio of extracted (mem)inductive flux and current. Inset on the top-right shows a picture of the experimental setup.

Extending the COMSOL simulation model to physically realize an ideal meminductor requires a mechanism to achieve current-induced relative motion between the core and the winding such that the direction of motion only changes when the polarity of the current signal changes, resulting in a phase difference of 90˚ between displacement and current. An experimental setup exploiting the interaction between a pair of neodymium permanent magnets and an electromagnet has been conceived to serve this objective: the magnetic poles on the winding switch whenever the current polarity changes which in turn reverses the direction of force between the permanent magnets and the winding. A time variation in the instantaneous inductance of the winding can be introduced by partially filling its core volume with a ferromagnetic material. As shown in Fig. 2a, the motion of the winding for the negative half-cycle of current results in gradually smaller winding volumes being occupied by the ferromagnetic core thus resulting in lower values of inductance (Supplementary Video-1). The motion is reversed during the positive half-cycle as the winding returns to its starting position as shown in Fig. 2b resulting in progressively higher values of inductance. The simulated magnetic field pattern (Supplementary Video-2) at the beginning of each run is shown in Fig. 2c, d. Also, the winding can be brought to a stop at any desired position (and by extension, inductance) by turning off the current and since this position does not change unless externally disturbed, the element designed possesses non-volatile memory in the form of a continuum of non-volatile inductance states.

Commercially available axially magnetized ring-type magnets with maximum remanent flux density of 14,800 G have been used as permanent magnets in this work. Two air core windings, W-1 and W-2, with stand-alone inductances of 50 mH and 150 mH, respectively have been fabricated from AWG-42 copper formvar and used as electromagnets to generate the results reported. Quasi-static inductance measurements have been performed on an LCR meter at a frequency of 1 kHz, AC amplitude of 10 mV, and DC bias voltage of 0 V and the results for W-1 for different positions of the winding relative to the core are shown in Fig. 2e. These measurements reveal a gradual change in inductance as the overlap between the winding and the core changes while the inductance plateaus on either side of this transition region.

The results reported in this section have been obtained by sourcing a sinusoidal current signal with an amplitude of 15 mA and frequency of 8 Hz through the winding W-1 and measuring voltage. The absence of an obvious phase difference between sourced current and measured voltage in Fig. 2f and the apparent single-valued linear behavior with a slope of 628 Ω in Fig. 2g show that resistive behavior dominates the electrical response of the element, as expected from COMSOL simulations. As shown in Fig. 2h,i, the total flux calculated as the time integral of the measured voltage remains unipolar and results in an ellipse when plotted as a function of current, thus reiterating the resistive behavior of the element at this frequency. However, extracting the inductive component of voltage by subtracting resistive voltage from the total voltage, followed by calculation of extracted flux as described in Eqs. (10) and (11), respectively, reveals hidden meminductive behavior.

10 $$\begin{array}{c}{v}_{L}\left(t\right)={v}_{measured}\left(t\right)-\left[{i}_{sourced}\left(t\right)*628 \Omega \right]\end{array}$$

11 $$\begin{array}{c}{\Phi }_{L}\left(t\right){\left.\right|}_{t={t}_{0}}={\int }_{0}^{{t}_{0}}{v}_{L}\left(t\right)dt \end{array}$$

Figure 2j shows the extracted inductive voltage as a function of time and its phase difference with the current being close to 90° confirms inductive behavior. The small spikes at the inductive voltage peaks can be attributed to the zero resistive voltage due to the sourced current being zero at these instants and the total voltage thus being very small and comparable to the electrical noise floor of the system. A difference between the positive and negative peak heights results in a distorted ellipse when *v*_*L*_ is plotted as a function of i. A linear inductor results in a perfect ellipse on the v-i plane, and a non-linear inductor, a distorted ellipse with an asymmetry about the i = 0 line. However, an ideal meminductor results in a distorted ellipse with an asymmetry about the v = 0 line, thereby signifying the existence of two distinct values of instantaneous inductive reactance for a given value of current. This behavior can be noticed in Fig. 2k, with the maximum excursions of the voltage on the positive and negative side being 0.114 V and 0.154 V, respectively. This confirms existence of meminductance ordinarily rendered invisible by a much more dominant resistive component.

A winding not under the influence of external magnetic fields remains at rest and the flux calculated as the product of quasi-static inductance and sourced current agrees with the values obtained through the time integral of the measured voltage. However, the presence of permanent magnets in the vicinity of the electromagnet influences its strength, thereby resulting in its flux being considerably higher than when at rest. Hence, for a winding in motion, it is necessary to define instantaneous dynamic inductance, calculated as the ratio of dynamic flux and current at any instant. Figure 2l, m show the extracted flux and the instantaneous dynamic inductance, L_inst_ as functions of time and current, respectively. Extracted flux denotes the (mem)inductive flux, Φ_L_ calculated as a time integral of the extracted inductive voltage and L_inst_ is calculated as the ratio of Φ_L_ and i at any instant. L_inst_ calculation for |i| < 5 mA becomes unreliable and results in unrealistically large values, with the denominator in Φ_L_/i being close to zero and hence, needs to be extrapolated from its values elsewhere. In Fig. 2l, it can be noticed that the extracted flux shares its zero-crossing points with the sourced current, while its positive and negative peaks lie on either side of those of current. Also, a close to 90° phase difference can be observed between L_inst_ and current, in agreement with the discussion from “Generalized mathematical description of circuit elements” section.

The extracted flux, when plotted as a function of current, results in a pinched hysteresis curve with a twist about the pinch point at the origin as shown in Fig. 2m^20^. L_inst_ plotted as a function of current reveals interesting behavior with L_inst_ momentarily decreasing between points A and B before increasing, while the polarity of current remains unchanged. As explained in “Generalized mathematical description of circuit elements” section, this pattern corresponds to generic behavior of the element rather than ideal. The physical origins of such behavior can be attributed to inertial motion of the winding causing it to continue in its previous direction of motion for a short duration even after a reversed polarity of current forces a reversal in the direction of the force. Also, the magnitude of current being low immediately following a reversal in polarity results in the force acting on the winding being low, thus contributing to considerable time elapsing before the force becomes strong enough to decelerate the winding to rest and accelerate it in the opposite direction. This generic behavior is further analyzed in “Position of the element realized on Chua’s periodic table” section.

## Frequency dependence and fingerprints of a meminductor

**Figure 3:**
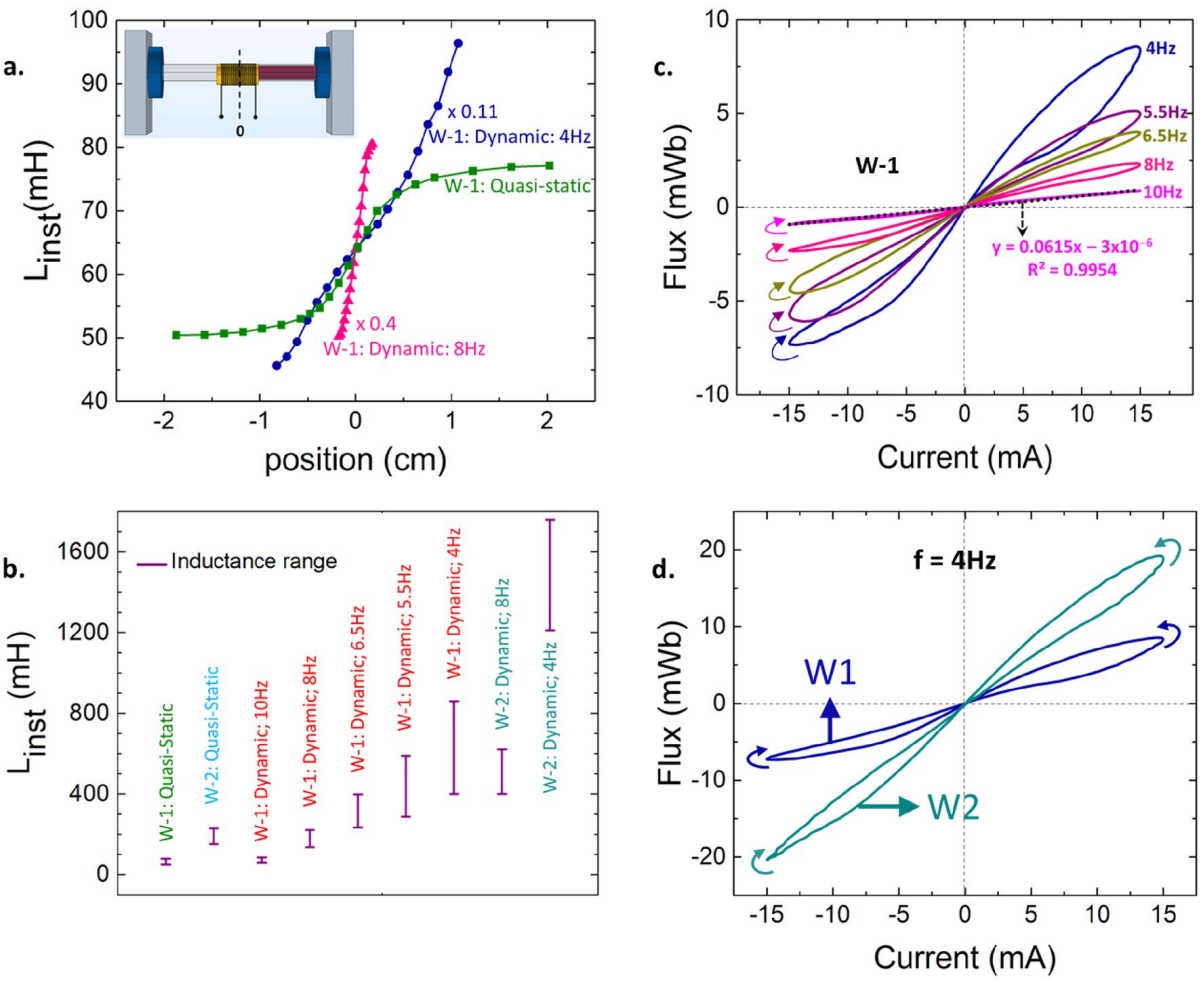
Frequency dependence: (**a**) Quasi-static and dynamic inductance (scaled down) measurements on W-1 as a function of the position of the winding relative to the core; zero-reference position in the setup shown in the inset. (**b**) Inductance ranges of W-1 and W-2 for quasi-static and dynamic measurements at different frequencies. Winding displacement decreases as the frequency increases resulting in the dynamic inductance measurements at high frequencies converging onto the quasi-static value. (**c**) The three fingerprints of a meminductor exhibited by the designed element. (**d**) Comparison of the pinched hysteresis curves obtained from the two windings fabricated, W-1 and W-2.

The maximum displacement of the winding increases with an increase in the amplitude of the current input and/or a decrease in the frequency, with low frequencies inducing the largest motion and high frequencies resulting in negligible movement. This is a direct consequence of the half-cycles lasting longer for lower frequencies, thus allowing more time for the winding to move in a certain direction before reversing. A larger displacement results in a smaller separation between the winding and the permanent magnets, thus resulting in larger dynamic flux and thereby, larger dynamic inductance. The dynamic inductance measurements for W-1 initially positioned with its center coinciding with the edge of the core are shown in Fig. 3a for sinusoidal current signals of frequencies of 4 Hz and 8 Hz and amplitude of 15 mA. The winding displacement (peak-to-peak) has been measured to be ~ 2 cm at 4 Hz and ~ 0.3 cm at 8 Hz thus resulting in the dynamic inductance being significantly greater at 4 Hz. As the frequency is increased, the dynamic inductance decreases until it converges onto the quasi-static value at that particular position once the winding displacement becomes negligible (Supplementary Video-3). The range of inductance values for W-1 and W-2 for different frequencies has been shown in Fig. 3b with the dynamic inductance of W-1 found to converge onto the quasi-static value as the frequency approaches 10 Hz.

Frequency dependence of the behavior of a memristor as captured by Leon Chua’s formulation of the three fingerprints of a memristor can be extended to a current-sourced meminductor and summarized as follows^21^: (1) When driven by a bipolar periodic current signal, the device must exhibit a “pinched hysteresis loop” in the flux-current plane, assuming the response is periodic. (2) Starting from some critical frequency, the hysteresis lobe area must decrease monotonically as the excitation frequency increases, and (3) the pinched hysteresis loop should shrink to a single-valued function when the frequency tends to infinity. Figure 3c shows that the element realized displays all the three fingerprints of a meminductor, i.e., pinched hysteresis curve in the flux-current plane, a monotonically decreasing lobe area as the frequency of sourced current increases and the response tending toward linear, single-valued behavior as the frequency increases beyond 10 Hz. The physical mechanism of the frequency dependence of the lobe area is an extension of the frequency dependence of the maximum winding displacement. As the frequency approaches 10 Hz the winding displacement becomes negligible resulting in time-invariant instantaneous inductance and thus, linear inductive behavior. This is further evidenced by the slope of the linear flux-current plot at 10 Hz being 61.5 mH, in perfect agreement with the quasi-static inductance measurements of W-1 from Fig. 3a. Figure 3d shows the comparison of the pinched hysteresis curves obtained for W-1 and W-2 at a frequency of 4 Hz. While the curve profiles look similar, the maximum flux for W-2 reaches close to 20 mWb while that of W-1, about 8 mWb with the difference arising from different inductance values of the two windings.

A consequence of frequency dependence of displacement is that for low frequencies, the winding can hit one of the permanent magnets, come to an abrupt stop, and stay immobile until the current polarity switches. This corresponds to a sudden switch in inductance from its dynamic value to a considerably lower quasi-static value, thus resulting in an abrupt shift in the voltage measurements and subsequent flux calculations. Hence, setup parameters such as the distance between the magnets, and sweep parameters such as amplitude and frequency of the current signal must be chosen carefully to prevent the winding from hitting the magnets. Also, the winding motion not being exactly reproducible over multiple cycles causes the pinched hysteresis curves to not close themselves at the end of every cycle and instead drift away from the origin. Further study is needed to understand and stabilize the motion of the winding so that it returns to the same position at the end of every cycle. Vibrational noise and time variations of the series resistance can also contribute to the curves drifting away from the origin and need to be eliminated.

## Position of the element realized on Chua’s periodic table

**Figure 4:**
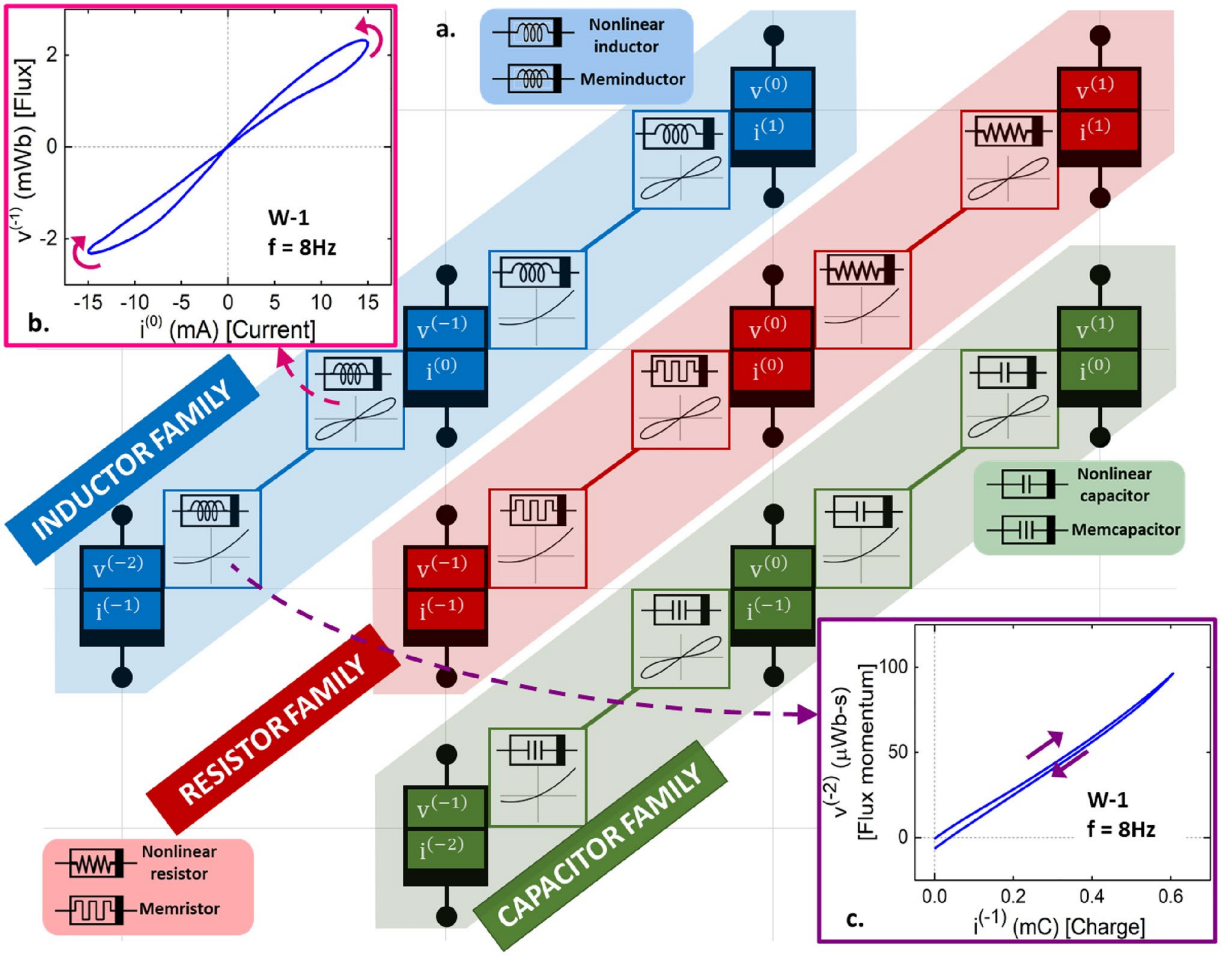
Position of the fabricated device on the periodic table of elements. (**a**) Leon Chua’s periodic table of two-terminal circuit elements with diagonals corresponding to the resistor, capacitor, and inductor families highlighted. The insets next to each (α, β) cell indicate the circuit elements with constitutive relationship and pinched hysteresis behavior in the (v^(α)^ − i^(β)^) plane. Electrical symbols of the six known nonlinear circuit elements are shown alongside their respective diagonals. (**b**,**c**) The device fabricated in this work exhibits a pinched hysteresis response in the (v^(−1)^ − i^(0)^) plane thus confirming its meminductive properties (**b**), and a multi-valued response in the (v^(−2)^ − i^(−1)^) plane reveals that the device is a generic meminductor (**c**).

A comparison of (2) and (6) reveals symmetry in the relationships, with the only mathematical difference between an ideal nonlinear inductor and an ideal meminductor being the choice of the ordered triple. While an ideal meminductor displays pinched hysteresis behavior in the (v^(−1)^ − i^(0)^) plane, an ideal nonlinear inductor displays similar behavior in the (v^(0)^ − i^(1)^) plane. In fact, this symmetry can be extended to any arbitrary choice of α and β to theorize the existence of an ideal (α, β)-element which exhibits pinched hysteresis behavior in the (v^(α+1)^ − i^(β+1)^) plane and is described by a constitutive relationship involving only v^(α)^ and i^(β)^ variables, leading to the periodic table of circuit elements, originally conceived by Leon Chua, and recreated in Fig. 4 with emphasis on results obtained in this work.

The plane in which a two-terminal passive circuit element displays pinched hysteresis behavior can identify the element. As such, the existence of a pinched hysteresis curve in the (v^(α+1)^ − i^(β+1)^) plane allows the grouping of each of the three traditional circuit elements and its respective mem-element into different families: the resistor family defined by α = β, the capacitor family by α = β + 1 and the inductor family by α = β − 1. As discussed in “Generalized mathematical description of circuit elements” section, while both ideal and generic versions of an (α, β) element result in a pinched hysteresis curve in the (v^(α+1)^ − i^(β+1)^) plane, only an ideal element results in a single valued response in the (v^(α)^ − i^(β)^) plane in at least one variable with one of v^(α)^ and i^(β)^ being zero whenever the other becomes zero.

The insets next to each (α, β) cell in Fig. 4a indicate the elements resulting in the respective response in the (v^(α)^ − i^(β)^) plane. For example, a pinched hysteresis curve in the (v^(0)^ − i^(0)^) plane corresponds to a memristor, while a single-valued non-linear behavior in the same plane represents an ideal nonlinear resistor. A pinched hysteresis curve in the (v^(−1)^ − i^(0)^) plane as shown in Fig. 4b serves to uniquely identify the element realized in this work as a meminductor. Further, Fig. 4c shows the response in the (v^(−2)^ − i^(−1)^) plane as being multi-valued in both the variables with the zero-crossing points of v^(−2)^ and i^(−1)^ not necessarily coinciding, thus qualifying the element as a generic meminductor rather than ideal. This result is in agreement with the discussion from “Experimental setup, results, and discussion” section, with the deviation from ideal behavior and onset of generic behavior explained as a consequence of inertial motion of the winding.

The deviation from ideal behavior raises questions on the physical realizability of any ideal two-terminal passive circuit element. Since there must be an inadvertent delay- regardless of how small- between a change in the sourcing function and a resultant change in the transfer function for any element, the transfer function becomes multi-valued in the source variable. For example, in a p–n junction diode- a supposedly ideal non-linear resistor- a change in the sourcing function, i.e., current (or voltage) needs to result in the diffusion of minority carriers across the space charge region before manifesting as a change in the instantaneous resistance (or conductance), thereby introducing a non-zero time delay- and thereby a non-zero phase difference- between the source variable and the transfer function. Hence, the i − v response of a p–n junction diode would in fact be memristive rather than resistive with the area of the lobes of the pinched hysteresis curve being potentially miniscule but non-zero, nevertheless. This line of discussion, while appearing to lend support to the arguments that an ideal memristor is not physically realizable^22,23^, in fact generalizes the idea to all ideal non-linear elements. The degree of deviation from ideality can vary depending on the timescales involved: negligibly small for a p–n junction diode due to short diffusion times of charge carriers and a lot more pronounced in the meminductor realized due to macroscopic displacements of the winding.

## Conclusions

Using the phase difference between the transfer function and the independent state variable to distinguish between an element and its mem-versions provides a new experimental perspective to aid the physical realization of any two-terminal circuit element. The element realized in this work has been shown to bear the three fingerprints of a meminductor and thus prove the physical evidence of meminductance, albeit overshadowed by a more dominant resistive component. The next step would be to make the series resistance less dominant, so that the element realized would truly be a meminductor without the need to extract hidden meminductive behavior. Operating the element in a cryogenic environment below the superconducting temperature of the winding appears the most feasible technique to eliminate series resistance in the configuration discussed. At room temperature, combating series resistance would require strengthening the inductive component through higher frequency operation; hence, replacing electromechanical means of varying instantaneous inductance with electronic phenomena is worth pursuing. Also, several existing physical systems such as solenoid plungers and audio speaker systems share similarities in operation principle with the meminductor described in this work and thus need further work dedicated to closer examination for meminductive properties.

## Supplementary Information

Supplementary Information 1.

Supplementary Information 2.

Supplementary Information 3.

Supplementary Information 4.

Supplementary Information 5.

## Data Availability

The raw data collected during this study is available in Supplementary Material. Further data resulting from analysis is available from the corresponding author upon reasonable request.
