# Supplementary material for: Physical evidence of meminductance in a passive, two-terminal circuit element
Source: Sci Rep. 2023 Feb 1;13:1817. doi: 10.1038/s41598-022-24914-y (PMC9892601; doi:10.1038/s41598-022-24914-y)
Supplement: Supplementary file 1 — Supplementary Information 1. [file 41598_2022_24914_MOESM1_ESM.docx]

**Supplementary Information**

**S.1 Generalized mathematical description of two terminal circuit elements**


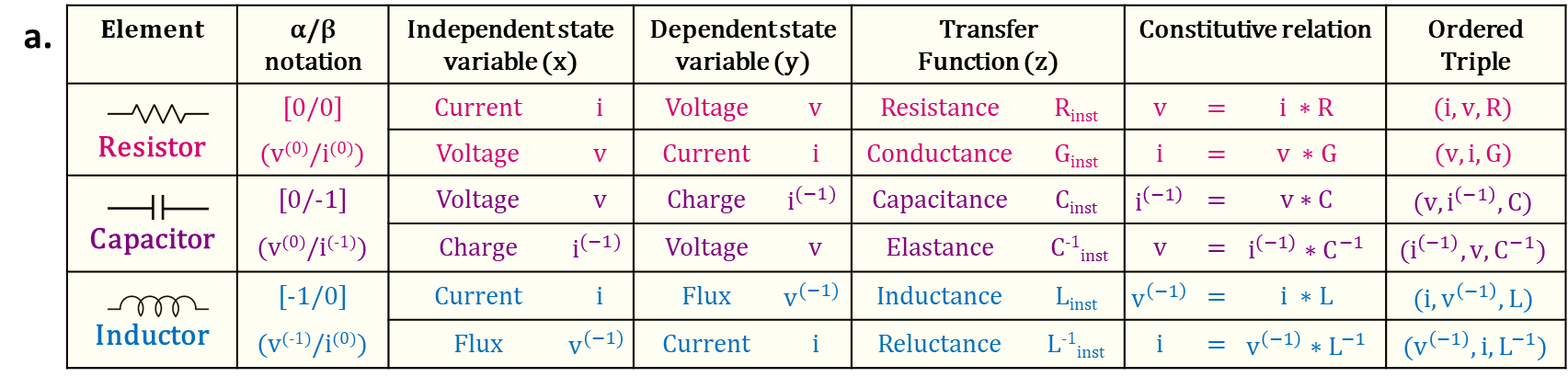

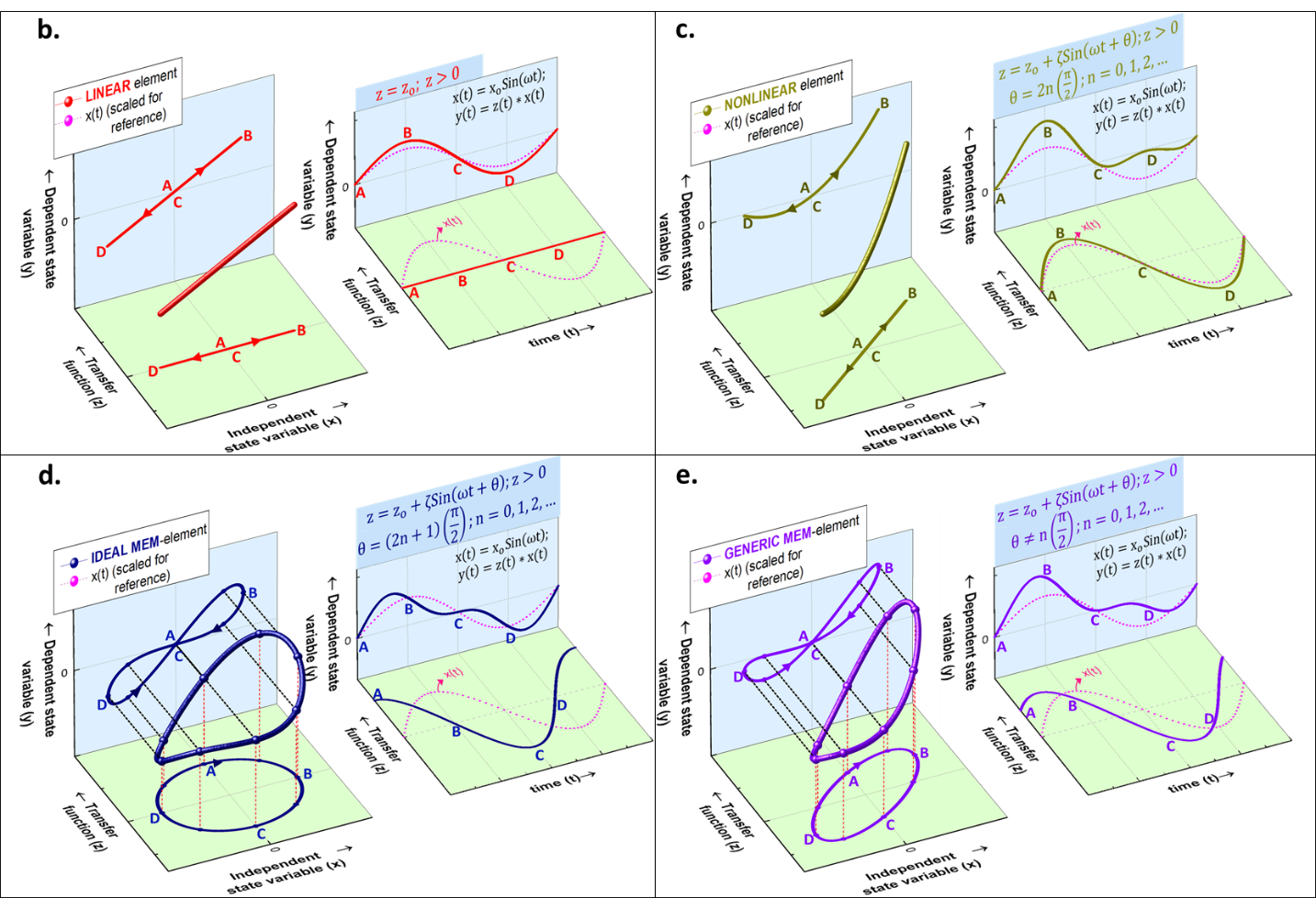


**Supplementary Fig. 1: Generalized mathematical description of two-terminal circuit elements.** **a)** Choice of the independent state variable, x(t), the dependent state variable, y(t), and the transfer function, z(t) to result in the three fundamental circuit elements (six ordered triple combinations) and their respective mem-versions. **b-e)** Time dependence of the state variables $\bar{\boldsymbol{s}}(t)$ results in a time-varying z(t). For a sinusoidally varying x(t), a constant z(t) yields a linear element **(b)**, whereas a variation in z(t) with a sinusoidal fit with the phase difference between x(t) and z(t) being even multiples of $\frac{\pi}{2}$ results in an ideal non-linear element **(c)**, a phase difference of odd multiples of $\frac{\pi}{2}$ results in an ideal mem-element **(d)**, and any other phase difference results in a generic mem-element **(e)**.

The discussion presented in the main text considers the (i, v^(-1)^, L) ordered triple and describes current and flux as the independent and dependent variables respectively in the mathematical development of an inductor and a meminductor. However, these elements can also be described using the (v^(-1)^, i, L^-1^) triple and the resulting mathematical descriptions for flux-sourced versions of a nonlinear inductor, a generic meminductor, and an ideal meminductor are given in (S1), (S2), and (S3), respectively.

$$\begin{aligned} i^{\left( 0 \right)} = L_{inst}^{-1}\left( v^{\left( -1 \right)} \right)*v^{\left( -1 \right)} \#\left( S1 \right) \end{aligned}$$

$$\begin{aligned} i^{\left( 0 \right)}=L_{inst}^{-1}\left( s^{-1}(t) \right)*v^{\left( -1 \right)} \#\left( S2.1 \right) \end{aligned}$$

$$\begin{aligned} \mathrm{where} \frac{ds^{-1}}{dt}=f\left( s^{-1}(t),v^{\left( -1 \right)} \right)\#\left( S2.2 \right) \end{aligned}$$

$$\begin{aligned} i^{\left( 0 \right)}=L_{inst}^{-1}\left( v^{\left( -2 \right)} \right)*v^{\left( -1 \right)} \#\left( S3.1 \right) \end{aligned}$$

$$\begin{aligned} i^{\left( -1 \right)}=L_{\left( -1 \right)_{inst}}^{-1}\left( v^{\left( -2 \right)} \right)*v^{\left( -2 \right)} \#\left( S3.2 \right) \end{aligned}$$

**S.2 Series resistance swamping meminductance**


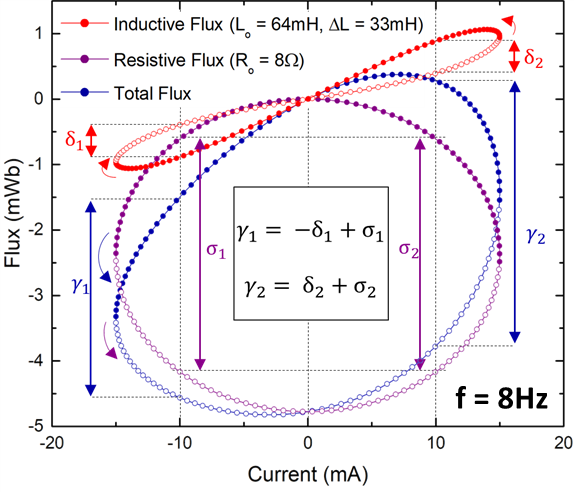


**Supplementary Fig. 2: Mechanism of series resistance swamping meminductance:** illustrated for a winding driven by a sinusoidal current signal, i(t) with I_o_= -15mA and f = 8Hz. Φ_T_, represented by the sum of a right-handed ellipse, Φ_R_, and a pinched hysteresis curve, Φ_L_, results in a distorted ellipse whose width is less (greater) than that of Φ_R_ in the left (right) half, where Φ_R_ and Φ_L_ have opposite (same) directions.

The three flux components, i.e., inductive flux, Φ_L_, resistive flux, Φ_R_, and total flux, Φ_T_ are all multivalued in current. Hence, the width of each component can be defined as the numerical difference between the two possible values of each at any given value of current, labelled in Supplementary Figure-2 as δ, σ, and γ, respectively. γ is numerically greater (less) than σ in the first (third) quadrant since the directions of Φ_L_ and Φ_R_ are the same (opposite). However, for any value of current, the absolute difference between σ and γ is numerically equal to δ.

**S.3 Comsol simulation details**


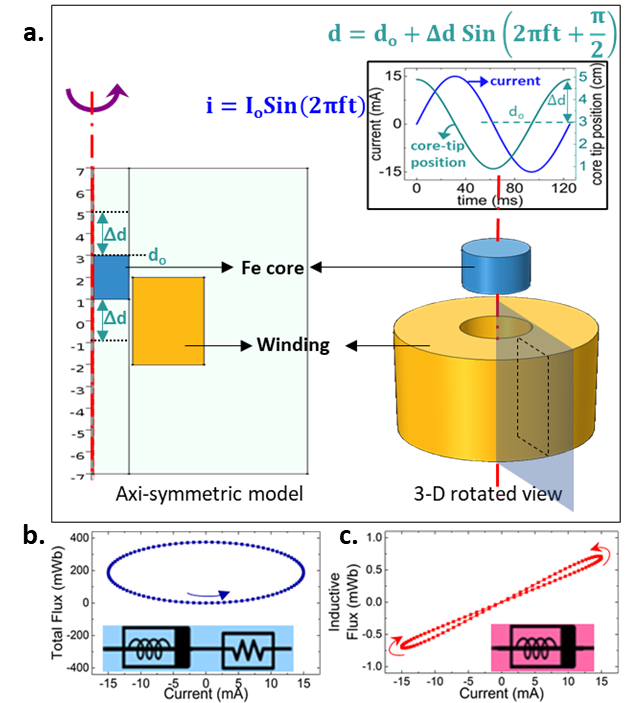


**Supplementary Fig. 3: Simulation model: a)** Axi-symmetric COMSOL model developed for simulating a current sourced winding with a movable ferromagnetic core; shown along with a 3-D rotated view. Winding parameters have been chosen to result in inductance and series resistance of the real winding fabricated. Inset: Core displacement and current defined mathematically to have a phase difference of 90^o^, **b)** Total flux calculated as the time integral of the voltage measured across the winding; elliptical profile reveals dominant resistive behavior, **c)** (Mem)inductive flux extracted by subtracting resistive flux from the total flux; pinched hysteresis characteristic confirms buried meminductive response.

An axi-symmetric COMSOL model of a winding with a movable ferromagnetic core has been developed such that relative motion between the winding and the core can be mathematically defined. As shown in Supplementary Figure-3(a), ‘d_o_’ represents the mean position of the tip of the movable core and ‘Δd’ denotes its displacement amplitude. The configuration in the “rest position” has been shown with the winding enclosing half of the core. Although any arbitrary phase difference can be introduced between i(t) and d(t) during simulation, ideal meminductive response can be generated by forcing a phase difference of 90^o^ as shown in the inset of Supplementary Figure-3(a). The results shown in Supplementary Figures-3(b, c) correspond to Δd, I_o_, and f values of 2cm, 15mA, and 8Hz, respectively. The winding parameters have been chosen to result in an inductance of 64mH with the core in the rest position and a series resistance of 628Ω has been used to replicate the measured inductance and resistance of the winding fabricated.
