# Supplementary figures and images for: Physical evidence of meminductance in a passive, two-terminal circuit element
Source: Sci Rep. 2023 Feb 1;13:1817. doi: 10.1038/s41598-022-24914-y (PMC9892601; doi:10.1038/s41598-022-24914-y)

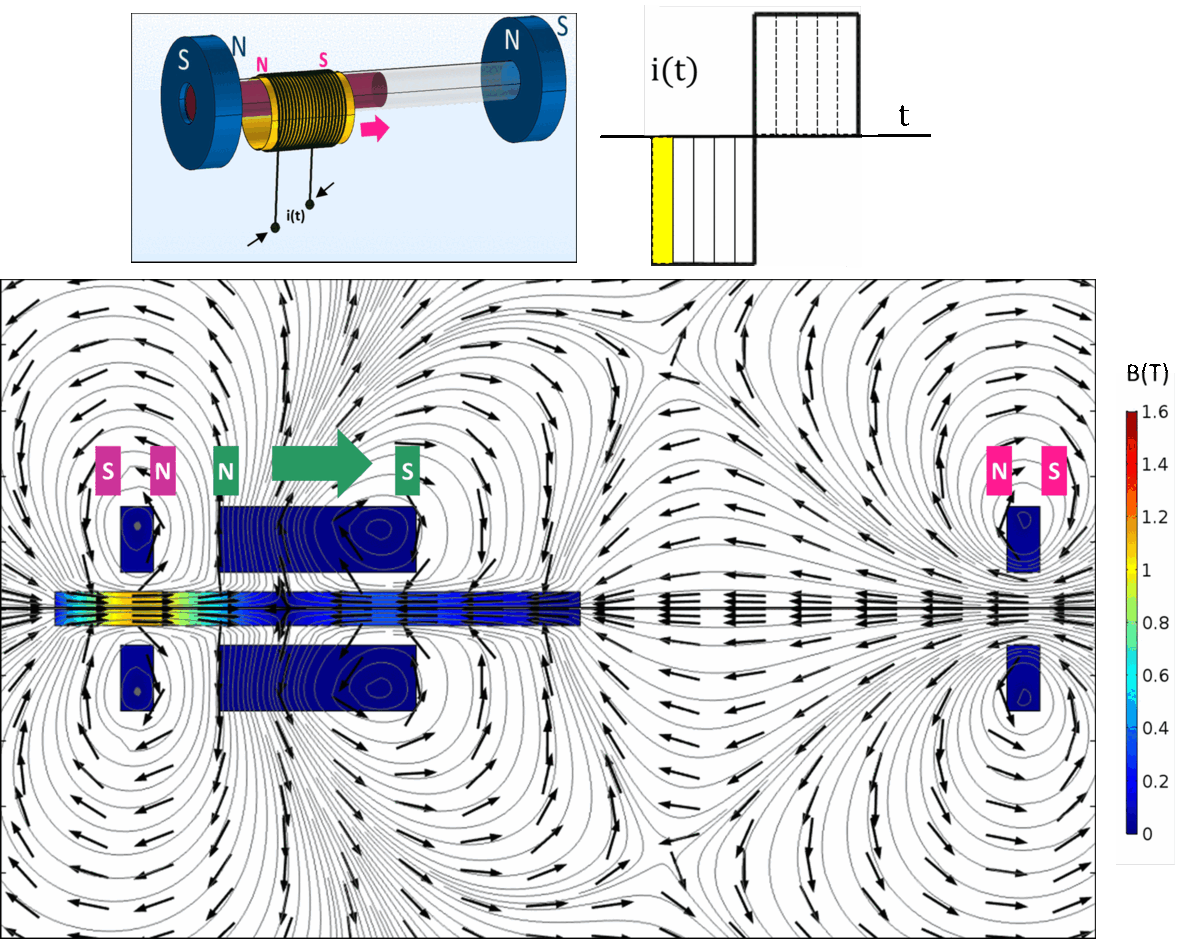

Supplement: Supplementary file 3 — Supplementary Information 3. [file 41598_2022_24914_MOESM3_ESM.gif]
